# Splénomégalie multi-nodulaire révélatrice d’une tuberculose multifocale à localisation splénique et vertébrale: à propos d’un cas

**DOI:** 10.11604/pamj.2021.40.230.32257

**Published:** 2021-12-16

**Authors:** Asmaa N´khaili, Mariama Jarti, Marj Zouhour Haida, Meryem Aouroud, Adil Ait Errami, Sofia Oubaha, Zouhour Samlani, Khadija Krati

**Affiliations:** 1 Service de Gastroentérologie, Centre Hospitalier Universitaire Mohammed VI Marrakech, Marrakech, Maroc,; 2 Laboratoire de Physiologie, Faculté de Médecine et de Pharmacie de Marrakech, Marrakech, Maroc

**Keywords:** Splénomegalie multinodulaire, mal de Pott, tuberculose extrapulmonaire, cas clinique, Multinodular splenomegaly, Pott´s disease, extrapulmonary tuberculosis, case report

## Abstract

La tuberculose splénique et le mal de Pott sont deux entités rares, notamment chez un sujet immunocompétent. Nous rapportons le cas d'une femme immunocompétente de 57 ans qui a présenté un tableau de douleur de l´hypocondre gauche atypique évoluant depuis 3 mois, associée à une paraparésie des 2 membres inférieurs d´installation progressive. Les données de laboratoire n'ont fourni aucune information spécifique pour le diagnostic, à part les résultats du QuantiFERON qui étaient positifs. La tomodensitométrie abdominopelvienne a révélé une splénomégalie avec de multiples lésions nodulaire hypodenses dans la rate. L´ imagerie par résonance magnétique (IRM) médullaire a montré un aspect de spondyldiscite de l´étage D10-D11 avec des collections épidurales et paravertébrales responsable d´une compression médullaire, le GeneXpert dans le prélèvement osseux était positif avec la présence d´un granulome centré par une nécrose caséeuse à l´étude histologique, le diagnostic de la tuberculose multifocale a été retenu.

## Abstract (en)

Splenic tuberculosis and Pott´s disease are two rare entities, in particular in immunocompetent people. We here report the case of a 57-year-old immunocompetent woman presenting with atypical pain in the left hypochondriac region evolving over the last 3 months, associated with lower limb paraparesis with progressive onset. Laboratory data did not provide any specific information regarding diagnosis, except for positive response to the QuantiFERON Test. Abdominal and pelvic computed tomography (CT) scan showed splenomegaly with multiple nodular, hypodense lesions in the spleen. Spinal cord magnetic resonance imaging (MRI) showed spondyldiscitis at the D10/D11 level with epidural and paravertebral collections responsible for medullary compression. GeneXpert assay on bone biopsy was positive and histological examination objectified granuloma characterized by the presence of central caseous necrosis. The diagnosis of multifocal tuberculosis was retained.

## Introduction

La tuberculose est une maladie infectieuse à transmission interhumaine liée au bacille de Koch (BK). C´est un problème majeur de santé publique dans le monde entier, en dépit des efforts fournis de lutte antituberculeuse [1]. La tuberculose splénique reste parmi les aspects rares de la tuberculose hématopoïétique des organes profonds, souvent associée aux autres localisations (pulmonaire, iléo-caecale...). La tuberculose splénique occupe une place importante dans les préoccupations des gastroentérologues, des hématologues et des chirurgiens; c´est une manifestation moins courante mais importante de la tuberculose abdominale, sa prévalence augmente avec la survenue de la VIH, même dans les pays à forte endémie, c´est une localisation rare. Nous rapportons le cas d´une tuberculose splénique et vertébrale, chez une patiente immunocompétente.

## Patient et observation

**Information de la patiente**: patiente S.A. âgée de 57 ans connue hypertendue sous amlodipine10 mg/jr, sans notion de contage tuberculeux, qui a été admise pour bilan étiologique de douleur de l´hypochondre gauche à type de pesanteur évoluant depuis 3 mois, suivi par l´apparition d´un déficit moteur partiel associé à des paresthésies des 2 membres inférieurs d´installation progressive depuis 1 mois.

**Résultats cliniques**: l´examen clinique a montré que la patiente était apyrétique avec une altération de l´état général, une énorme splénomégalie d´aspect bosselé à la palpation, sans signes d´hypertension portale ni d´adénopathies périphériques; pour l´examen neurologique, la marche était en steppage, Romberg et station debout difficile à évaluer, vu le déficit, les réflexes ostéotendineux étaient vifs, diffus, polycinétiques aux deux membres inférieurs, Babinski bilatéral, le reste de l´examen somatique était par ailleurs normal.

**Figure 1:**
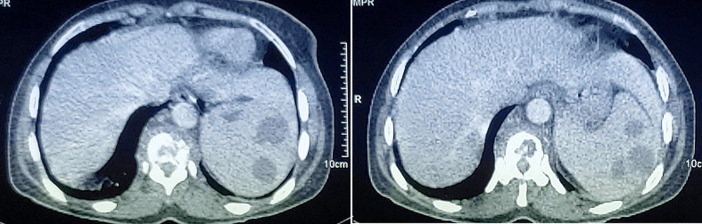
coupes scanographiques axiales à l’étage abdominal montrant une rate augmentée de taille, siège de multiples lésions ovalaires hypodenses

**Figure 2:**
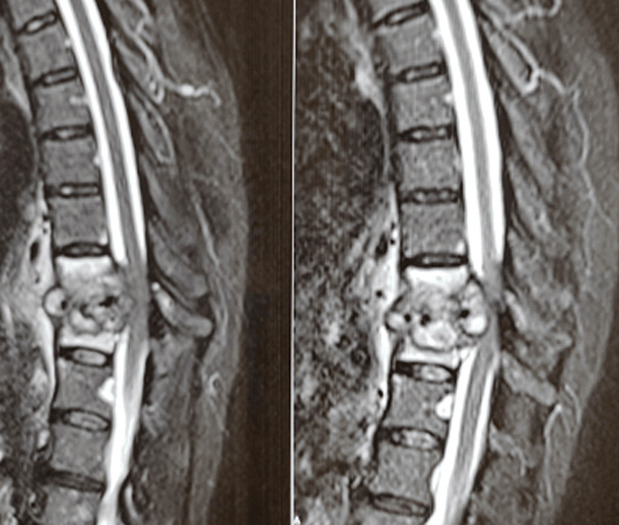
coupes sagitales de l’IRM médullaire montrant un aspect de spondyldiscite de l’étage D10-D11 avec des collections épidurales et para-vertébrales responsables d’une compression médullaire

**Démarche diagnostique**: une numération formule sanguine a donné les résultats suivants: numération leucocytaire = 4210/µL, hémoglobine = 11.9g/dL, les plaquettes étaient à 290000/mm^3^, sans autres anomalies biologiquement notable. La radio thorax était normale; recherche de BK et GeneXpert dans les crachats étaient négatives, le dosage du QuantiFERON était positif, la sérologie HIV s´est révélée négative et le dosage des marqueurs tumoraux était normal. Le dosage des marqueurs tumoraux était normal. La tomodensitométrie thoraco-abdominopelvienne a objectivé l´existence d´une atteinte disco-verterbrale dorsale de D10-D11 compliquée d´une collection prévertebrale associé à une splénomégalie multinodulaire, siège de multiples lésions nodulaires au nombre de neuf, hypodenses, bien limités de forme ovalaire, sans adénopathie décelable (Figure 1). L´imagerie par résonnance magnétique médullaire a montré un aspect de spondyldiscite de l´étage D10-D11 avec des collections épidurales et para-vertébrales-responsables d´une compression médullaire (Figure 2). La ponction à l'aiguille fine de la rate n'a pas été réalisée car techniquement irréalisable. La patiente a été par la suite référée au service de neurochirurgie où elle a bénéficié d´une décompression de la moelle et des racines et l´évacuation de ces collections. Un prélèvement osseux par voie transpediculaire a été réalisée en regard du D10. L´étude histologique a objectivé une lésion granulomateuse épithéliogiganto-cellulaire avec des cellules géantes à type de Langerhans, centré par endroit par une nécrose caséeuse, le GeneXpert au niveau du prélèvement osseux était positif.

**Intervention thérapeutique et suivi**: le diagnostic d´une tuberculose multifocale a été retenu devant les arguments bactériologiques et histologiques, la patiente a été mise sous traitement antituberculeux pendant 9 mois (2RHZE/7RH) avec une bonne évolution clinique et biologique.

## Discussion

La tuberculose extrapulmonaire représente environ 16% de tous les cas de tuberculose en 2019 selon l´Organisation Mondiale de la Santé [2]. La tuberculose splénique peut exister au sein d´un tableau d´une atteinte diffuse en particulier hépatoganglionnaire et médullaire dite hématopoïétique, elle représente environ 1% de toutes les tuberculoses et 10% des formes extrapulmonaires [3]. Pour La tuberculose musculo-squelettique c´est environ 10% des cas de tuberculose extrapulmonaire et 1 à 5% de tous les cas de tuberculose [4]. Ces localisations extra-pulmonaires sont très souvent évocatrices d´une immuno-dépression. L´atteinte de la rate nous semble plutôt répondre à une dissémination à partir d´un foyer d´infestation initiale, ancien ou récent, symptomatique ou le plus souvent méconnu et négligé. Le mécanisme de dissémination est lymphatique ou hématogène. L´agent bactérien en cause de cette affection est le plus souvent le bacille de Koch bovin ou humain [4].

La tuberculose splénique peut se manifester par des aspects cliniques variables mais non spécifiques. L´amaigrissement, la fièvre et l´anémie sont les manifestations les plus courantes [5,6]. L´hépatomégalie peut être présente, c´est le cas de la majorité des tuberculoses abdominales [7]. Une évolution rapide, souvent mortelle, avec cachexie, fièvre, hémorragie ou surinfection peuvent exister dans certaines formes dites malignes. Pour poser le diagnostic des formes pseudo-tumorales. L´échographie et la tomodénsitométrie, constituent une étape primordiale en termes de la tuberculose splénique. Les images hétérogènes avec des foyers hypodenses en rapport avec de la nécrose caséeuse rendent le diagnostic de tuberculose difficile. Surtout qu´elles peuvent simuler d´autres affections telle une lésion tumorale maligne primitive ou secondaire [8,9]. Une preuve bactériologique peut s´avérer ainsi nécessaire par la mise en évidence des Bacilles Acido-Alcolo Résistants (BARR) après la coloration spéciale de Ziehl-Neelsen et une mise en culture systématiquement des prélèvements. La confirmation n´est que bactériologique parfois histologique mais cette dernière n´est pas spécifique, la recherche de la tuberculose pulmonaire associé est systématique, à travers la réalisation de nombreux prélèvements au niveau des expectorations spontanées ou induites, le tubage gastrique, les aspirations bronchiques et le lavage broncho-alvéolaire [1,10].

Cependant la non spécificité des signes cliniques, biologiques et radiologiques rend l'examen histologique indispensable pour le diagnostic de la tuberculose splénique, c´est surtout par le biais de la biopsie à l'aiguille de la rate ou la splénectomie. La ponction à l'aiguille fine est un outil précieux, avec une sensibilité de 88 % et une spécificité allant jusqu'à 100 % [11]. Dans notre cas, la ponction à l'aiguille fine n'a pas été réalisée car techniquement irréalisable. Quant à la spondydiscite à tuberculose, autrement dit, le mal de Pott. c´est la maladie granulomateuse la plus courante de la colonne vertébrale, qui se caractérise par le fait d´être chronique et lentement progressive; la confirmation se fait à travers l´isolement Mycobacterium de la tuberculose ou l'identification des granulomes dans un échantillon obtenu à partir des vertèbres atteints [11,12]. Les études d'imagerie sont importantes pour la détection des maladies, principalement la tomodensitométrie (TDM) et l'imagerie par résonance magnétique (IRM) qui depuis 1987 ont permis de détecter la maladie à une phase prédestructive [11,13]. L´IRM a permis de faciliter le diagnostic précoce, par la mise en évidence d´un bilan d´extension loco-régionale, de visualiser des abcès intra et extracanalaire, elle a ainsi un très grand intérêt à la fois diagnostic et pronostique [14]. Le traitement de la tuberculose splénique est avant tout médical, basé sur l´association de trois antibiotiques: la rifampicine (R), l´isoniazide (H) et le pyrazinamide (Z) [15].

Le traitement médical proposé est 2HRZE/4HR pour l´Organisation Mondiale de la Santé [16], 2HRZE/7HR pour d´autres [17]. Le traitement chirurgical n´est indiqué qu´en présence de symptômes neurologiques de compression, concernant la spondylodiscite [18]. Les différentes techniques proposées visent à décomprimer la moelle et les racines, l´évacuation des abcès volumineux, le rétablissement de la statique rachidienne et conforterle diagnostic par une confirmation histologique [19], comme le cas de notre patiente. En l'absence de signes neurologiques, l'évolution insidieuse des lésions disco-vertébrales du mal de Pott retarde son diagnostic, d´où l´intérêt d´entamer précocement le traitement anti-bacillaire associé à une prise en charge neurochirurgicale et radio-conventionnelle grâce à l'apport de l'IRM, dans l´intention d'améliorer le pronostic [20].

## Conclusion

La tuberculose splénique dans sa forme pseudotumorale est une affection rare dont le diagnostic de certitude est histologique et/ou bactériologique, l´atteinte isolé de la rate pose un problème diagnostic. L´association à un autre foyer de tuberculose extrapulmonaire, autrement dit la spondyldiscite, dans cette situation a permis de poser facilement le diagnostic et d´entamer le traitement antibacillaire, avec une bonne amélioration sans recours à d´autres preuves histologiques.
